# Rib fractures after reirradiation plus hyperthermia for recurrent breast cancer: Predictive factors

**DOI:** 10.1007/s00066-016-0946-3

**Published:** 2016-02-08

**Authors:** Sabine Oldenborg, Christel Valk, Rob van Os, Bing Oei, Jack Venselaar, Paul Zum Vörde Sive Vörding, Adriënne van Randen, Hans Crezee, Geertjan van Tienhoven, Coen Rasch

**Affiliations:** 1 Department of Radiation Oncology, Z1-215, Academic Medical Center, University of Amsterdam, Meibergdreef 9, P.O. Box 22660, Amsterdam, The Netherlands; 2 Institute Verbeeten (BVI), Tilburg, The Netherlands; 3 Department of Radiology Academic Medical Center, University of Amsterdam (AMC), Amsterdam, The Netherlands

**Keywords:** Reirradiation, Hyperthermia, Recurrent breast cancer, Local control, Toxicity, Rebestrahlung, Hyperthermie, Rezidivierender Brustkrebs, Lokale Kontrolle, Toxizität

## Abstract

**Background:**

Combining reirradiation (reRT) and hyperthermia (HT) has shown high therapeutic value for patients with locoregional recurrent breast cancer (LR). However, additional toxicity of reirradiation (e.g., rib fractures) may occur. The aim of this study is to determine the impact of potential risk factors on the occurrence of rib fractures.

**Patients and methods:**

From 1982–2005, 234 patients were treated with adjuvant reRT + HT after surgery for LR. ReRT consisted typically of 8 fractions of  4 Gy twice a week, or 12 fractions of  3 Gy four times a week. A total of 118 patients were irradiated with abutted photon and electron fields. In all, 60 patients were irradiated using either one or alternating combinations of abutted AP electron fields. Hyperthermia was given once or twice a week.

**Results:**

The 5-year infield local control (LC) rate was 70 %. Rib fractures were detected in 16 of 234 patients (actuarial risk: 7 % at 5 years). All rib fractures occurred in patients treated with a combination of photon and abutted electron fields (*p* = 0.000); in 15 of 16 patients fractures were located in the abutment regions. The other significant predictive factors for rib fractures were a higher fraction dose (*p* = 0.040), large RT fields, and treatment before the year 2000.

**Discussion and conclusion:**

ReRT + HT results in long-term LC. The majority of rib fractures were located in the photon/electron abutment area, emphasizing the disadvantage of field overlap. Large abutted photon/electron fields combined with 4 Gy fractions increase the number of rib fractures in this study group. However, as these factors were highly correlated no relative importance of the individual factors could be estimated. Increasing the number of HT sessions a week does not increase the risk of rib fractures.

## Abstract (de)

**Hintergrund:**

Der kombinierte Einsatz von Rebestrahlung (reRT) und Hyperthermie (HT) zeigt eine hohe Wirksamkeit bei Patienten mit lokoregional rezidiviertem Brustkrebs (LR). Jedoch können zusätzliche toxische Effekte von reRT (z. B. Rippenfrakturen)auftreten. Ziel war es, potentielle Risikofaktoren für die Entstehung von Rippenfrakturen zu untersuchen.

**Patienten und Methoden:**

Von 1982–2005 wurden 234 Patienten nach LR-Operation mit adjuvanter reRT + HT behandelt. Die reRT wurde üblicherweise mit 8 × 4 Gy 2/Woche oder 12 × 3 Gy 4/Woche durchgeführt. Mit sich anschließenden Photonen- und Elektronenfeldern wurden 118 Patienten bestrahlt. Eine Bestrahlung mit einem oder mehreren versetzten AP-Elektronenfeldern erhielten 60 Patienten. Die HT wurde 1- oder 2-mal pro Woche verabreicht.

**Ergebnisse:**

Die lokale Kontrollrate (LC) betrug 70% nach 5 Jahren. Insgesamt wurden bei 16/234 Patienten Rippenfrakturen festgestellt (Risiko 7% nach 5 Jahren). Alle Frakturen traten bei Patienten auf, die mit einer Kombination von Photonen- und Elektronenfeldern behandelt wurden (*p* = 0,000); bei 15/16 Patienten waren Frakturen in der Anschlussregion lokalisiert. Weitere wichtige prädiktive Faktoren für Rippenfrakturen waren eine höhere Fraktionierungsdosis (*p* = 0,040), große RT-Felder sowie eine Behandlung vor dem Jahr 2000.

**Diskussion und Schlussfolgerung:**

Durch reRT + HT lässt sich eine langfristige LC erzielen. Die Mehrheit der Rippenfrakturen war in den Photonen-/Elektronenanschlussregionen lokalisiert, was den Nachteil von Feldüberlappungen bekräftigt. Große angrenzende Photonen-/Elektronenfelder kombiniert mit 4-Gy-Fraktionen erhöhen die Zahl von Rippenfrakturen in dieser Studiengruppe. Da die verschiedenen Faktoren stark korrelierten, konnte keine relative Bedeutung der Einzelfaktoren ermittelt werden. Mehr HT-Sitzungen pro Woche erhöhen das Risiko für Rippenbrüche nicht.

Locoregional recurrence (LR) after mastectomy or breast conservation predicts a poor outcome in patients with breast cancer [1–3]. Hyperthermia (HT), the elevation of tumor temperature to 40–45 °C, is a well-established radio- and chemotherapy sensitizer [4, 5]. Several phase III trials demonstrated a significant increase of complete response rates and duration of local control when hyperthermia was added to radiotherapy for locoregional recurrent breast cancer in previously irradiated areas [6, 7]. The Dutch National guidelines therefore adopted the combination of reirradiation (reRT) + HT as standard of care for recurrent breast cancer in previously irradiated area [8].

The reported incidence of rib fractures after primary breast cancer treatment varies from < 1 to 19 % [9–13], depending on detection methods. The incidence decreased to < 2 % in more recent years, due to the improvement of radiation techniques [9, 10, 13].

After reRT for recurrent breast cancer, toxicity, like rib fractures, are likely to occur as well. Although several authors report on the incidence of rib fractures [9–13], data on the risk and significant cause(s) of rib fractures after reRT + HT in previously irradiated area are scarce. The current study aims to retrospectively evaluate the number and location of rib fractures after adjuvant reRT + HT in 234 patients treated in two Dutch clinical centers for locoregional recurrent breast cancer in previously irradiated area after macroscopic complete resection or a clinically complete remission after chemotherapy. The impact of potential risk factors on the occurrence of fractures is also investigated.

## Methods and materials

### Patients

Data were collected from all patients with locoregional recurrent breast cancer in areas previously irradiated with curative intent, treated with reRT and HT at the Academic Medical Center (AMC) and at the Ben Verbeeten Institute (BVI). Patients treated from 1982 onward, the year clinical hyperthermia started in the Netherlands, up to 2006 were included to enable long-term follow-up. A total of 234 (AMC: 152, BVI: 82) patients received ReRT + HT as an adjunct to surgery or chemotherapy.

Data were collected from the radiotherapy and hyperthermia patient charts. In case of missing follow-up data, questionnaires were sent to referring specialists, and/or general practitioners. X-rays and CT scans were collected from patients who were reported to have rib fractures on any follow-up visit after reRT + HT. Imaging was performed when patients suffered from pain or other symptoms or when disease progression was suspected. The number and location of the fractures were assessed by one of the researchers (C.V.) and confirmed by a radiologist (A.R.)

All patients received previous high dose radiation, overlapping with the current reRT field. Of the patients, 42 % were treated for previous locoregional recurrent disease using surgery, radiation, systemic therapy, or a combination of treatment modalities, before start of reRT + HT.

For the current recurrence episode, 225 patients had a macroscopically complete surgery and 9 patients a clinically complete remission (cCR) after chemotherapy. Characteristics of the current disease episode and potential risk factors for rib fractures are summarized in Table 1.

**Table 1:** Patient and treatment characteristics

Current episode	BVI	AMC	Total
Median FU time	56 (0.6–151) months	43 (0.7–207) months	47 (0.6–207) months
Median age at current treatment	55 (35–78) years	53 (28–89) years	54 (28–89) years
Menopausal status^a^			
Post	54 (82 %)	107 (79 %)	161 (80 %)
Median TI surgery—reRT^b^	2 (0.7–5) months	2 (0.8–8) months	2 (0.7–8) months
Median TI primary RT—reRT^c^	66 (12–404) months	67 (6–553) months	66 (6–553) months
Median primary RT dose^d^	50 (49.2–55.8) Gy	50 (30–92.7) Gy	50 (30–92.7) Gy
Local or regional pre-RT boost^e^	1 (1.2 %)	9 (5.9 %)	10 (4.6 %)
Median dose pre-reRT boost^f^	11.2 Gy	13.8 (8.9–25.1) Gy	12.50 (8.9–25.1) Gy
Median total pre-reRT dose^g^	50 (49.2–61.4) Gy	50 (30–92.7) Gy	50 (30–92.7) Gy
Surgery (R0/R1)^h^/Chemotherapy (cCR)			
Salvage mastectomy	35 (43 %)	66 (44 %)	101 (44 %)
Chest wall resection (CWR)	4 (5 %)	12 (8 %)	16 (7 %)
Wide resection/partial CWR	3 (4 %)	4 (3 %)	7 (3 %)
Local excision	36 (44 %)	63 (42 %)	99 (43 %)
Chemotherapy	4 (5 %)	5 (3 %)	9 (4 %)
Median reRT field size^i^	2.3 (0.5–6.0) dm^2^	4 (0.8–36) dm^2^	3.5 (0.5–36.0) dm^2^
ReRT dose^b^			
10/20/25 × 2 (20/40/50) Gy	1 (1 %)	2 (2 %)	3 (1 %)
6/10/11 × 3 (22.2/37/40.7) Gy	3 (3 %)	1 (2 %)	4 (2 %)
12 × 3 (44.4) Gy	77 (94 %)		77 (33 %)
5/6 × 4 (23.3/35.2) Gy		3 (1 %)	3 (1 %)
8 × 4 (46.9) Gy	1 (1 %)	139 (91 %)	140 (60 %)
10 × 4 (58.6) Gy		7 (4 %)	7 (3 %)
Median reRT dose^k^	44.4 (20–46.9) Gy	46.9 (22.2–58.6) Gy	46.9 (20–58.6)Gy
Median total dose (pre-reRT + reRT)^l^	64.4 (40–68.9) Gy	66.9 (40.6–95.7) Gy	66.7 (40–95.7)Gy
Systemic treatment^m^	42 (51 %)	83 (55 %)	125 (53 %)
Chemotherapy^n^	18 (22 %)	26 (17 %)	44 (19 %)
Hormone therapy^o^	33 (40 %)	67 (54 %)	100 (44 %)
Median electron-energy^p^	9 (4–15) MeV	8 (6–15) MeV	8 (4–15) MeV
Median photon-energy^q^	6 (6–15) MV	6 (5–14) MV	6 (5–15) MV

*TI* time interval, *FU* follow-up, *BVI* Ben Verbeeten Institute, *AMC* Academic Medical Center

^a^BVI: missing for 16 patients, AMC: missing for 17 patients

^b^BVI: missing for 4 patients, AMC: missing for 9 patients

^c^BVI: missing for 1 patient, AMC: missing for 2 patients

^d^Prior to reRT + HT; AMC: missing for 2 patients

^e^BVI: for 2 patients, AMC: data missing for 15 patients

^f^In addition to the reRT + HT

^g^AMC: missing for 1 patient

^h^BVI: missing for 5 patients

^i^BVI: missing for 6 patients, AMC: missing for 20 patients

^j^BVI: missing for 1 patients, AMC: missing for 9 patients

^k^Dose in EQD2 excl. boost; BVI: missing for 1 patient, AMC: missing for 11 patients

^l^Pre-reRT local or regional boost overlapping the current reRT area given for previous recurrent disease

^m^Total dose boost in EQD2

^n^Total pre-reRT dose incl boost in EQD2

^O^in parenthesis dose in EQD2

^p^Dose in EQD2; BVI: missing for 1 patient, AMC: missing for 1 patient

^q^Total reRT dose in EQD2, incl 40 % of the total pre-reRT dose; BVI: missing for 2 patients, AMC: missing for 12 patients. α/β = 2.3

### Treatment

#### Radiotherapy

**Fig. 1:**
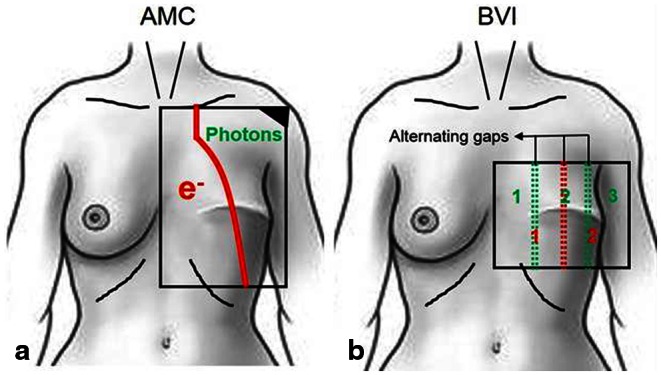
Standard chest wall radiation technique. **a** One anterior–posterior electron field abutted to one anteroposterior–posteroanterior photon field. **b** Alternating use of 2 or 3 abutted anteroposterior electron fields separated by either 1 or 2 small gaps

All AMC patients were irradiated using a standard schedule of 8 fractions of 4 Gy given twice a week to a total dose of 32 Gy. At BVI, the standard reRT schedule consisted of 12 fractions of 3 Gy given four times a week to a total dose of 36 Gy. For large target areas, abutted photon and electron fields were used at AMC (Fig. 1a). In general, the chest wall or mastectomy area up to the dorsal axillary fold was considered the target area.

At BVI, large target areas were treated with a combination of 2–3 alternating abutted AP electron fields. Fields are separated by gaps, creating a number of different fields. Alternatingly, either 2 or 3 fields will be irradiated simultaneously. The distance between the alternating gap locations was adjusted for each individual patient to minimize both underdosage and overdosage (Fig. 1b).

#### Hyperthermia

HT was given once a week at AMC and twice a week at BVI, starting within 1 h after radiotherapy. Heat was induced electromagnetically, using externally applied contact flexible microstrip applicators, operating at 434 MHz [14]. Treatment fields covered the entire target area. The use of one single applicator was sufficient to cover the entire target area for all patients. For all patients, temperatures were measured with thin flexible multisensory thermocouple probes on the skin and, if feasible or preferable, invasively using a flexible subcutaneous catheter. The target temperature was 41–43 °C for 1 h, following a preheating phase of approximately 15 min. Applied power was adjusted to the desired temperature distribution without exceeding the maximum normal tissue temperatures (45 °C) or patient tolerance.

### Endpoints and data analysis

#### Rib fractures

Rib fractures were graded according to the National Cancer Institute’s Common Terminology Criteria for Adverse Events, version 3.0 as bone fractures were not included in version 4. The rib fracture incidence was calculated by the actuarial method of Kaplan and Meier from the start of reRT + HT to the date of first rib fracture notification. Patients without rib fractures were censored at date of last follow-up. The location of rib fractures for each individual patient were marked on two standard left/right chest wall CT scans relatively to the radiation fields. For one patient locating rib fractures was not possible because volumetric data were lost. As this patient was reported with radiation-induced rib fractures, we did include this patient in the statistical analysis and EQD2 calculations.

#### Local control and survival

Both local control (LC) and survival rate were calculated from the date of first re-irradiation fraction. Duration of LC and survival were analyzed by the actuarial method of Kaplan and Meier. Local failure was defined as in-field relapse. Patients dying with LC, or alive with continuing LC at last follow-up, were censored at the date of death or last follow-up, respectively.

#### Statistics

Statistical analysis was carried out using SPSS version 20 (SPSS Inc., Chicago, IL, USA). A univariable analysis was done for the occurrence of rib fractures, using patient, disease, and treatment related variables. A total of 14 variables with potential prognostic value were tested. Only variables available for at least 80 % of the population were included. The level of statistical significance was considered < 0.05 for all analyses. Analyses were carried out using the Cox regression test or the Fisher’s exact test, depending on the number of events. The continuous variables were checked for linearity by using spline regression curves and spline coefficients tested for nonlinearity; all continuous variables showed a linear relationship with the occurrence of rib fractures. A multivariable analysis was not deemed appropriate because of the small total number of events.

#### Equivalent dose at 2 Gy (EQD2)

The maximum possible EQD2 at the rib area was calculated for the two different reRT fraction doses and for the two different reRT techniques, using the linear-quadratic (LQ) model [15]:

$$\text{EQD2}=D\times [(d+\alpha /\beta )/2~Gy+\alpha /\beta )]$$

where *D* denotes the total reRT dose and *d* the dose per fraction. *α/β* was assumed to be 2.3 Gy for the ribs [15]. The maximum possible overdose on the ribs when using abutted electron/photon fields was calculated using the AMC planning system (Oncentra, v.4.3, Elekta). A standard CT scan from 2 breast cancer patients were used for simulating dose distributions and calculating the maximum possible overdose on the ribs. Calculations were done for perfectly abutted photon/electron fields (6 MV photons without bolus, 10 MeV electrons with bolus).

For alternating abutted electron fields with gaps in between, the maximum possible overdose on the ribs was calculated using Theraplan-Plus (MDS Nordion, Kanata, Canada).

## Results

### Rib fractures

**Fig. 2:**
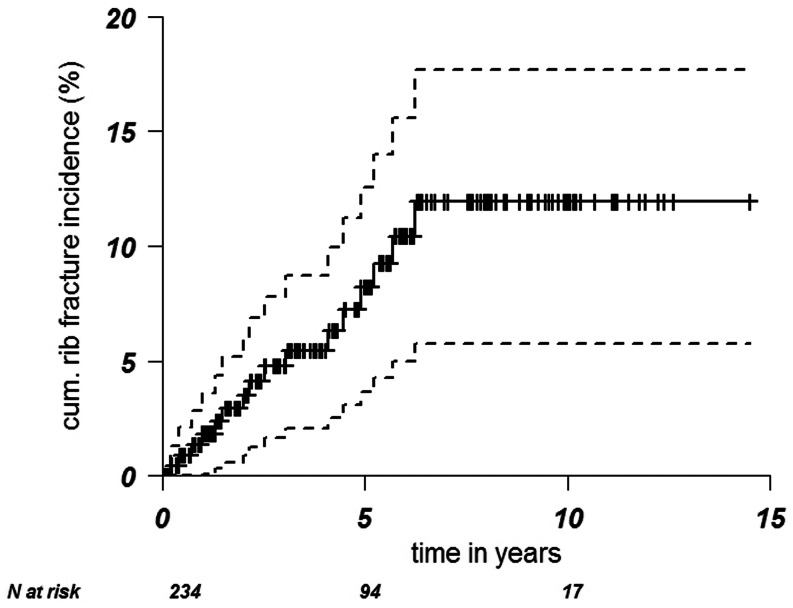
Actuarial rib fracture incidence. ---- = 95 % confidence interval, + = censored, ǀ= at risk

**Fig. 3:**
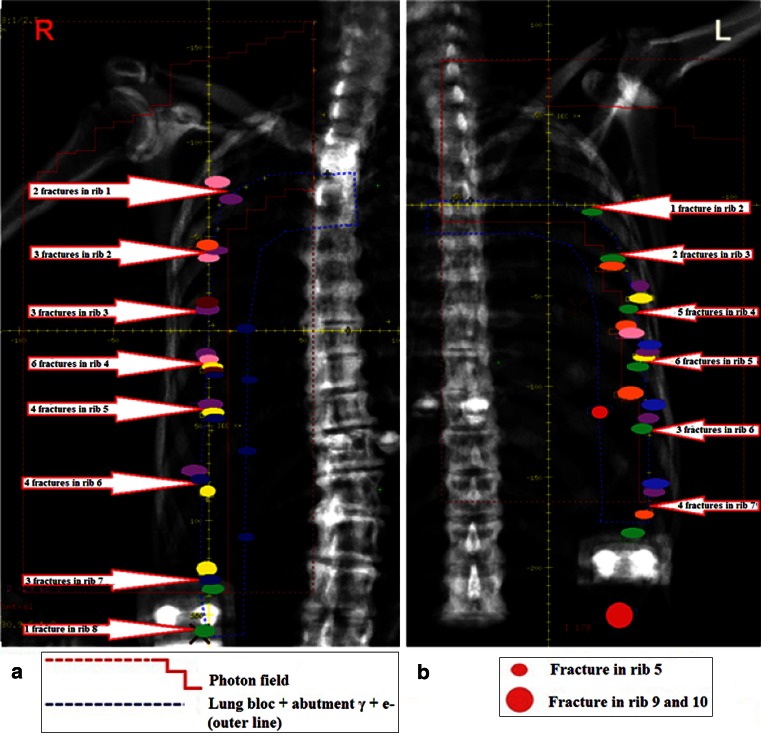
Chest wall CT-sans of two random breast cancer patients showing the AMC treatment plan setup: **a** Left chest wall. **b** right chest wall, different patient. Each color represents rib fractures of an individual patient. In red fractures in rib 5, 9 and 10 are indicated for a patient which were not associated with abutment regions.*γ* photon field. *e* ^*−*^ electron field

In 16 of 234 patients (7 %) 1–8 fractures occurred after reRT + H, whereby 15 of those patients were treated at AMC and 1 at BVI. The maximum risk increased to 12 % at 6.2 years (Fig. 2). Five patients had asymptomatic rib fractures (grade 1), 7 patients had symptomatic fractures (grade 2), and the other 4 patients had grade 3 fractures for which hyperbaric oxygen treatment was indicated. The location of rib fractures is shown in Fig. 3a and b. All fractures occurred in the reRT area. The number of rib fractures per patient ranged from 1–8. Rib 4 was most frequently fractured. In all 15 AMC patients, fractures were located in the photon/electron abutment area anterolateral in the patient. The chart from the patient who lacked radiologic data also reported the fractures to be in this abutment area. In one patient fractures occurred both in reRT abutment area and in the area of overlap with the previously irradiated parasternal field. The only BVI patient with rib fractures was treated with tangential photon fields + 2 abutted electron fields. Although this patient did have rib fractures, the fractures were not located in the abutment regions. The group of 17 patients treated with other RT schedules (Table 2) did not show rib fractures.

**Table 2:** Univariable Cox regression for rib fracture incidence

Covariate institute	*P*-value BVI	*P*-value AMC	*P*-value Total	HR total
ReRT technique^a^:	0.173	0.128	0.000	^a^
e^−^ + γ : other				
reRT fraction dose:	NA	NA	0.033	9.049
3 Gy (BVI): 4 Gy (AMC)				
ReRT dose:	0.922	0.915	0.312	1.079
Continuous (in EQD2)				
Total ReRT dose	0.831	0.749	0.628	1.028
Continuous (in EQD2)				
reRT field size^b^:	0.740	0.271	0.047	3.612
≤ 2.3: > 2.3 dm^2^				
Year of treatment:	0.673	0.037	0.040	0.344
≤ 1999: ≥ 2000				
TI current surgery—reRT:	0.592	0.113	0.104	0.537
< 2: ≥ 2 months				
Current surgery:	0.602	0.669	0.505	1.437
Local excision: Mastectomy				
Chemotherapy treatment^c^:	0.596	0.154	0.127	2.203
No: Yes				
Age:	0.205	0.409	0.252	0.975
Continuous				
Electron-energy:	0.311	0.447	0.535	1.089
Continuous (4–15 MeV)				
TI primary RT—ReRT:	0.747	0.258	0.356	0.606
< 65: ≥ 65 months				
Menopausal status:	0.668	0.211	0.494	1.682
Pre: Post				
Hormone treatment^c^:	0.648	0.148	0.444	0.680
No: Yes				
Photon- energy:	NA	0.827	0.820	0.955
Continuous (5–15 MV)				
Total Pre-reRT dose (in EQD2):	0.250	0.638	0.605	0.966
Continuous				

*TI* time interval, *HR* razard ratio; *e* ^*−*^ *+ γ*; abutted photon/electron fields, *NA*  not applicable, *BVI* Ben Verbeeten Instituteγ, *AMC* γAcademic Medical Centerγ

^a^Fisher’s exact test; no HR calculable

^b^Missing values: 7 %

^c^Previous treatment or in addition to the reRT + HT

The 5 year infield LC rate was 70 % with an overall survival rate of 60 %.

### Prognostic factors

The results from these analyses are presented in Table 2. The covariates and their measure on entry in the analyses are shown in column 1. Four factors were found to significantly affect rib fracture incidence. The use of abutted photon/electron fields significantly contributed to the incidence of rib fractures compared to all other RT techniques. A fraction dose of 4 Gy resulted in a significantly 9-fold higher risk on rib fractures compared to 3 Gy. Smaller field sizes and year of treatment from 2000 and above reduced the risk on rib fractures. Pre-reRT dose, reRT dose and total dose in EQD2, not corrected for reRT technique, were not significantly related to the occurrence rib fractures.

### EQD2

The 12  fraction of  3 Gy schedule, used at BVI can result in a EQD2 of 44 Gy in the rib area, when part of the rib cage is located within the radiation field. The 8 fractions of  4 Gy schedule, used at AMC, would result in a slightly higher EQD2 of 47 Gy. Simulation results for perfectly abutted photon/electron fields showed the local overdose on part of the ribs to vary between 0 and 40 %. The maximum possible EQD2 on the ribs with a 40 % overdose would then be 87 Gy for 8 fractions of  4 Gy. This is 74 % higher than the TD 5/5 (EQD2 = 50 Gy; the dose likely to produce rib fractures in 5 % of patients within 5 years after radiation exposure)[16].

When using 12 fractions of  3 Gy and alternating electron fields separated by gaps (4.5–9 MeV, with 0.5 or 1 cm bolus) the local calculated overdose on the ribs varied from 0–10 %. The EQD2 on the ribs with a 10 % overdose is 51.6 Gy, exceeding the TD 5/5 by 3 %.

## Discussion

We found a difference in rib fracture rate of 1 vs. 10 % for the different treatment institutes (BVI vs. AMC). The AMC fracture rate is higher than the < 2 % rate reported for primary radiation [9, 10, 13]. Our reported fracture rate might actually be higher than reported here due to the retrospective character of our study.

The main differences in reRT treatment between both institutes were number of HT fractions, reRT field size, -schedule, and -technique. In all, 76 % of BVI patients were treated with only one electron field or 2–3 abutted alternating electron fields (Fig. 1) and a lower reRT fraction dose was used, whereby 73 % of AMC patients were treated with APPA abutted photon/electron fields, and in general reRT fields were larger.

Univariable analyses showed the use of abutted photon/electron fields to significantly affect the risk on rib fractures in agreement with the results from Fig. 3. ReRT fraction dose, field size, and year of treatment also significantly affected the occurrence of rib fractures. Multivariable analyses could not be done, because of the small number of events. However, our findings are in agreement with known risk factors for primary high dose irradiation: hypofractionation, low machine energy, large target volume, older age, female gender, postmenopausal status, surgical procedure preceding irradiation, and adjuvant simultaneous or sequential chemotherapy [11, 12].

The separate effects of abutted fields, fraction dose, and field sizes could not be estimated because of the high correlation of these factors. As the other tested factors were equally distributed between the two institutes, but not found significant, they were considered of lesser predictive value for rib fractures in our patient population. The significant impact of year of treatment on rib fracture occurrence may be related to the availability of improved RT techniques after 1999. In 2000 computer tomography-based diagnosis and RT planning allowed target areas to be delineated more precisely, reducing the risk of normal tissue damage. After the year 2000, the risk on rib fractures significantly decreased at AMC (*P* = 0.037, HR = 0.318), but not at BVI as the number of fractures observed at BVI was already low (1 %). The rib fracture rate for patients treated at AMC from 2000–2005 was still as high as 6 %, probably reflecting the unchanged presence of risk factors such as large abutted fields combined with high fraction dose.

Few studies have reported on rib fracture incidence with different radiation schedules, after reRT for recurrent breast cancer (Table 3). Comparison with those studies is difficult because most studies included very few patients, RT techniques differ and follow-up times are generally too short, as patients can develop fractures between 1 month and 5 years after treatment [12]. Some larger retrospective studies reported on rib fractures after using the same reRT schedule as used at AMC. A study by van der Zee et al. [17] reported on 8/134 patients (7 %) with bone necrosis or fracture. Another study, done by the same institute, did not report rib fractures for patients with resectable breast recurrences [18]. Several reasons might account for this discrepancy. First, only a minority of patients were irradiated with photon/electron fields (23 %). Second, only late grade 3 and 4 toxicities were reported, which included 5 patients who required treatment with necrotomy, reconstruction, and/or hyperbaric oxygen for osteoradionecrosis. Thus, the actual rib fracture rate was probably underreported in their third study. Several small sized studies show low frequencies (≤ 1 %) of rib fractures with hyperfractionation [19–23]. In general, higher fractions doses seemed to be related to higher rib fracture incidences, regardless of the total reRT dose. This is in agreement with results from randomized phase III trials comparing radiation schedules with fraction doses varying from 2.3 versus 3.9 to 2.7–3.2 versus 2 Gy. These results indicated that fraction dose could be a factor in the development of rib fractures above a threshold (approximately  3.2–3.9 Gy) even when the total dose is modest [10, 11, 13].

**Table 3:** Previous studies of external beam repeat chest wall irradiation for recurrent breast cancer using different ReRT schedules

Study	Median FU (months)	Patients (n)	Additional treatment	Total reRT- dose	reRT fraction- dose	Rib fractures	reRT technique electrons + photons
Li et al. 20	(6–179)	41	HT	Med 43.0 Gy	1.8–2.0 Gy	0 (0 %)	0 (0 %)
Deutsch 19	51.1	39	Surgery	50 Gy	2.0 Gy	0 (0 %)	0 (0 %)
Zee vd et al. 17	(1–2 years)	121	HT	32 Gy	4 Gy	8 (7 %)	?
Müller et al. 21	41	42	Surgery + HT	Med 60 Gy	1.8–2 Gy	1 (2 %)	0 (0 %)
Wahl et al. 22	17	70	HT/CT	Med 48 Gy	1.8–2 Gy	1 (1 %)	4 (6 %)
Linthorst et al. 18	42	198	Surgery + HT	32 Gy	4 Gy	0 (0 %)	45 (23 %)
Wurschmidt et al. 23	13.7	29	± CT	Med. 50.4 Gy	1.6–2.5 Gy	1 (3 %)	?

*CT* chemotherapy, *HT* hyperthermia, *Med* median, *PRDR* pulsed reduced dose-rate radiotherapy, *FU* follow-up

Abutted fields are known to cause dosimetric problems as overlapping fields can result in a substantial local over- or underdose, especially at a depth correlating with the location of the ribs.The maximum physical overdose varies with patient anatomy, and photon/electron energy and can be even be further exceeded by human or mechanical errors. When combining 3 Gy fractions with alternating electron fields the risk of overdose is minimized.

Both qualitative and quantitative analyses of our patient group suggest that abutted photon/electron fields and a 4 Gy fraction dose increase the risk of rib fractures and might lead to the “double trouble” effect of (1) difference between calculated dose and actual dose at any point and (2) variation in biological effects with different fraction doses) when combined [24]. Restoration of normal tissue tolerance after low and moderate initial RT doses is on average no more than 60 % after 6 months, depending on tissue type [25]. Hence, for determining the optimum reRT schedule a number of parameters must be taken into account: the initial EQD2, volume treated, amount of overlap, additional treatments, and time interval between therapy courses [25].

Doubling HT fractions did not affect rib damage in our study population. This is in agreement with results from other HT studies that indicate that the number of HT sessions does not influence toxicity and that hyperthermia does not significantly affect overall toxicity when added to (re)RT [6, 7, 26, 27].

Our patients will be more prone to reRT damage as they received high dose irradiation and different kinds of systemic therapies in the past and reRT is preceded by surgery. Avoiding large abutted fields and reducing fraction dose might therefore also reduce overall toxicity. As a result of this study, RT techniques using only tangential photon fields or IMRT and lower fraction doses have been adopted at AMC in order to minimize the risk of problems with abutting fields.

## Conclusion

ReRT + HT results in long-term LC of 70 % after 5 years. In 7 % of patients, rib fractures occurred, the majority of which were located in the photon/electron abutment area, emphasizing the disadvantage of field overlap. Large abutted photon/electron fields combined with 4 Gy fractions increased the number of rib fractures in this study group. No relative importance of the individual factors could be estimated. Increasing the number of HT sessions a week does not increase the risk of rib fractures.
